# Supplementary material for: Members of the Legionella pneumophila Sde family target tyrosine residues for phosphoribosyl-linked ubiquitination
Source: RSC Chem Biol. 2021 Jul 29;2(5):1509–19. doi: 10.1039/d1cb00088h (PMC8496037; doi:10.1039/d1cb00088h)
Supplement: CB-002-D1CB00088H-s001 [file CB-002-D1CB00088H-s001.pdf]

Electronic Supplementary Material (ESI) for RSC Chemical Biology.
This journal is © The Royal Society of Chemistry 2021

Members of the *Legionella pneumophila* Sde Family Target Tyrosine Residues for Phosphoribosyl-Linked Ubiquitination

Mengyun Zhang[‡1,2], Joseph M. McEwen[‡3], Nicole M. Sjoblom[3], Kristin M. Kotewicz[1,2], Ralph R. Isberg[*1], Rebecca A. Scheck[*3]

[1]Department of Molecular Biology and Microbiology, Tufts University School of Medicine, Boston, MA, USA
[2]Graduate Program in Molecular Microbiology, Tufts University Graduate School of Biomedical Sciences, Boston, MA, USA
[3]Department of Chemistry, Tufts University, Medford, MA, USA
[‡]These authors contributed equally

*Corresponding emails: ralph.isberg@tufts.edu, rebecca.scheck@tufts.edu

Electronic Supplementary Information

Materials and Methods

General Materials	2
Protein Purification and Peptide Synthesis	2
Rtn4 Ubiquitination Using Two-step SdeC Bead Assay	3
HA-Ub Polymerization Assay and HA Peptide Ubiquitination Assays	3
Polyacrylamide Gel Electrophoresis and Western Blotting	3
FPLC Purification and Size Exclusion Chromatography	3
General Protocol for Trypsin Digestion of ADP-ribosylated Ub and Crosslinked Proteins	3
Mass Spectrometry	3

Supplementary Figures and Tables

**Figure S1**	HA-Ub polymerization is dependent on HA tag	5
**Table S1**	Mass error anlysis for HA-Ub xl-fragment	6
**Table S2**	Mass Error Analysis for Ub_{WT} + HA Peptide Phe-Variants xl-fragment.	7
**Table S3**	Mass Error Analysis for Ub_{WT} + HAY_{249S} xl-fragment	8
**Figure S2**	SdeC does not have an intrinsic preference for Ser over Tyr linkages	9
**Table S4**	Mass Error Analysis for Ub_{WT} + HA Peptide Ser-Variants xl-fragment	10
**Table S5**	Plasmids used in this study	11
**Table S6**	Peptides used in this study	11

**Supplementary References**	12

General Materials. All chemical reagents and solvents were of analytical grade, obtained from commercial suppliers and used without further purification unless otherwise noted.

Protein Purification and Peptide Synthesis. SdeC variants were purified as described previously.[1,2] *Escherichia coli* strain BL21 (DE3) strain carrying bacterial expression vector pQE-80L (N-terminal Poly His tag) with either full length SdeC WT or point mutants, each containing a C-terminal StrepII tag, was used for protein expression. Bacteria cultures were grown in 2X Yeast Tryptone broth (2xYT) overnight with 100 µg/ml ampicillin, back diluted and grown until A600 = 0.6, at which point 1mM IPTG (Sigma) was added for 4-6 hours to induce protein expression. Cells were collected by centrifugation, resuspended in 2% of the culture volume with lysis buffer (50mM NaH_2PO_4, 300 mM NaCl, 10 mM imidazole, pH 7.6), then subjected to 4-5 freeze-thaw cycles before sonication. Soluble proteins were purified from collected supernatants using Ni-NTA agarose resin (Thermo Fisher) followed by Strep-tactin resin (IBA) affinity chromatography according to manufacturer's protocol. Purified proteins were dialyzed into 20 mM Tris, 10 mM NaCl, pH 7.4 (1X ART buffer), and concentrated with Amicon Ultra Centrifuge filters (30K MWCO). Protein concentrations were determined by Bradford assay (Bio-Rad) using BSA as standard. Concentrated proteins were then mixed with 10% glycerol, aliquoted, and stored at -80°C. Purified GST-Rtn4 was purchased from MRCPPU. Human recombinant monomeric Ub and HA-Ub were purchased from Boston Biochem (R&D systems). All HA peptide variants were synthesized in Tufts University Core Facility. All plasmids and peptides in this study are further described in **Tables S5 & S6**.

Rtn4 Ubiquitination Using Two-step SdeC Bead Assay. *In vitro* Rtn4 ubiquitination assays were described previously.[1] For linking recombinant SdeC derivatives to agarose beads, Ni-NTA agarose resin (Thermo Fisher) was washed 3X with ART buffer and mixed with His_6-SdeC followed by incubation for 1 h at 4 °C. Then, the SdeC-bound agarose resin was washed 3X with 1X ART buffer before proceeding to the two-step bead assay. During preincubation, SdeC-bound agarose beads were added to 10 mM Ub and 100 mM ε-NAD in 1X ART buffer (SdeC final concentration 50 nM), and incubated for 1 h at 37 °C. After incubation, the beads were removed from reaction by spin filter columns (Pierce spin cups, paper filter that was pre-wet with 1X ART buffer). The supernatant containing modified Ub and excess NAD were mixed with 400 nM GST-HA-Rtn4 and fresh unbound SdeC variants, as indicated, then incubated for another hour at 37 °C. Reactions were terminated by addition of reducing loading buffer and boiling.

HA-Ub Polymerization Assay and HA Peptide Ubiquitination Assays. For HA-Ub polymerization assays, 1 µM HA-Ub was mixed with 100 µM Nicotinamide 1, N6-ethenoadenine dinucleotide (ε-NAD, Sigma) or β-Nicotinamide adenine dinucleotide (β-NAD, Santa Cruz Biotech/Sigma), and recombinant SdeC at final concentration of 20 nM. The reaction mix was incubated at 37 °C in 1X ART buffer for 2 h. For HA peptide ubiquitination assays, 12.5 µM HA derivative peptides were mixed with 10 µM monomeric Ub, and incubated with 20 nM SdeC and 250 µM β-NAD at 37°C in 1X ART buffer for 1 h. All reactions for gel analysis were terminated by either addition of reducing loading buffer and boiling,

or flash freezing in liquid N_2 for storage prior to further analysis. For samples analyzed by mass spectrometry (MS), excess salts and NAD were removed via buffer exchange, using either Illustra NAP-5 Columns (Cytiva Life sciences) or Amicon Ultra Centrifuge filters, 3K MWCO (MilliporeSigma), eluting in water.

Polyacrylamide Gel Electrophoresis and Western Blotting. After assays for HA-Ub crosslinking or pR-Ub modification, samples boiled in reducing SDS loading buffer were fractionated by sodium dodecyl sulfate polyacrylamide gel electrophoresis (SDS-PAGE). Assays using Ub as a substrate, as well as HA-Ub and HA-peptides, were fractionated on 15% gels, while the GST-Rtn4 reactions were fractionated on 5% gels followed by either Coomassie Brilliant Blue staining or silver staining. Silver stains were performed as described in manufacturer's protocol (Invitrogen SilverQuest Silver Staining kit). The mouse monoclonal anti-Ub antibody (mAb P4D1, 1:500) and anti-ethenoadenosine mAb (1G4; 1:500) were purchased from Santa Cruz Biotechnology. Anti-mouse IgG (H+L) (DyLight™ 800 4X PEG Conjugate) was purchased from Cell Signaling Technology (1:20,000). LI-COR Odyssey CLx was used for Western blot signal detection.

FPLC Purification and Size Exclusion Chromatography. After crosslinking reactions were complete, samples were concentrated 10X by lyophilization (Labconco Freezone12), then purified by FPLC with 1X ART buffer on a Superdex 200 10/300 GL column (flow rate 0.25 ml/min). 0.2 ml fractions from the HA-Ub monomer and dimer peaks were collected and combined based on SDS PAGE fractionation and silver staining.

General Protocol for Trypsin Digestion of ADP-ribosylated Ub and Crosslinked Proteins. Using Amicon® Ultra centrifugal filters (Millipore Sigma), intact protein reactions were concentrated and underwent buffer exchange into water, and were diluted into 1:1 *t*-butanol:water using a 3K MWCO. DTT was added to a final concentration of 3 mM. Reactions were then heated to 65 °C for 35 minutes and subsequently cooled to room temperature. Sequencing grade modified trypsin (Promega) was added for a final 1:25 protease:protein ratio. This digest solution was incubated at 37 °C overnight. The resulting solution of peptides was subjected to LC-MS/MS analysis without further purification.

Mass Spectrometry. For intact protein analysis, samples were injected onto a ZORBAX 300SB-C8 column (2.1 x 100 mm, Agilent) and eluted with a water:acetonitrile gradient mobile phase with 0.1% formic acid (0.400 mL/min; 5% - 80% over 24 min). The mass spectrometer was utilized in positive mode with a dual electrospray ionization (ESI) source. MS spectra were acquired using the following settings: ESI capillary voltage, 4500 V; fragmenter, 250 V; gas temperature, 350°C; gas rate, 12.5 L/min; nebulizer, 50 psig. Data was acquired at a rate = 5 spectra per second and scan range of 100 – 3000 m/z. Analysis was completed using Agilent MassHunter Bioconfirm (v. B.06.00).

Following tryptic digestion, peptide fragments were injected onto an AdvanceBio Peptide 2.7 µm column (2.1 x 150 mm, Agilent) and were eluted with the same gradient and flow rate as indicated above. MS

spectra were obtained using the same parameters as described above. MS/MS spectra were acquired using the following settings: ESI capillary voltage, 4000 V; fragmentor, 150 V; gas temperature, 325°C; gas rate 12 L/min; nebulizer, 40 psig. MS/MS was acquired at 1 spectra per second with a mass range of 300 – 3000 m/z. For all MS/MS acquisitions, targeted precursor ion selection, coupled with set collision energies were used. Full length precursor ion m/z's were calculated using ChemDraw. Precursors were identified *via* MS scans with the following stringency: medium isolation width, z=2-4, RT = 10 min, ΔRT = 2 min). After identification, precursor ions were subjected to iterative rounds of collision induced dissociation in the collision chamber and subsequent mass identification. MS/MS cycles scanned five ionization energies from 20-28 V, with a 2 V step between scans to sample various ionization events for each precursor ion. Following acquisition, all MS/MS spectra were combined for display. On average, targeted MS/MS acquisition yielded 80-140 spectra for each defined precursor ion.

Analysis was completed using multiple programs. Agilent MassHunter Bioconfirm (v. B.07.00) was used for generation of intact protein deconvolutions. Bioconfirm was also used to generate extracted ion chromatograms (EIC) and additional mass spectra. Exact masses were calculated using ChemDraw Prime (PerkinElmer, v. 16.0). ChemDraw also enabled identification of unique fragmentation patterns. Posttranslational modifications (PTMs) were identified using PEAKS Studio (v. 7.5) software. Utilizing PEAKS Search feature, preliminary analysis enabled 0.2 Da error tolerance for a tryptic digestion with non-specific cleavage allowed at either end of the peptides and up to 3 missed cleavages. Unspecified PTMs and common mutations were searched to match Ubiquitin or pR-HA-Ub sequences. For verification, new PTMs were created in the software and analysis repeated with greater stringencies to confirm MS/MS matching[3,4]. Mass error analysis was completed by matching observed fragment ions with predicted fragmentations generated in ChemDraw. Error is reported in ppm as well as amu. Figures created in Adobe Illustrator.

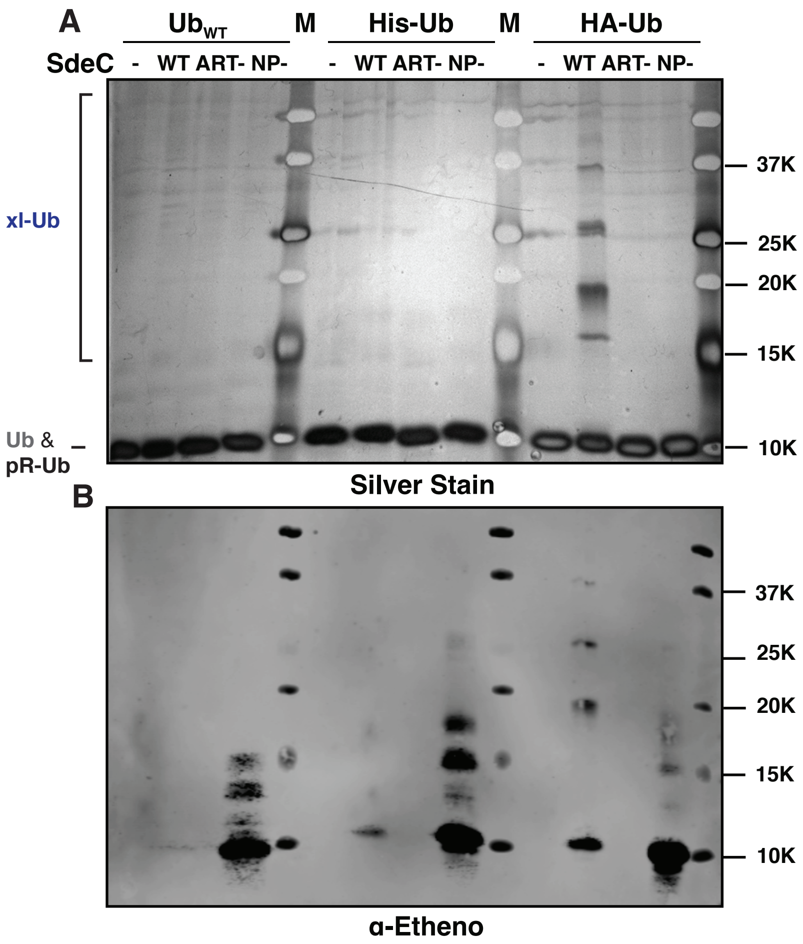

Figure S1. HA-Ub polymerization is dependent on HA tag. Ub$_{WT}$, His-Ub, HA-Ub were incubated with ε-NAD$^+$ and SdeC at 37 °C for 2 h, then fractionated by SDS-PAGE, followed by **A)** silver staining or **B)** western blot probing with α-ethenoadenosine antibodies.

Table S1. Mass Error Anlysis for HA-Ub xl-fragment.
Mass error analysis for identified and assigned fragment ions labeled in **Main Text Figure 3**.

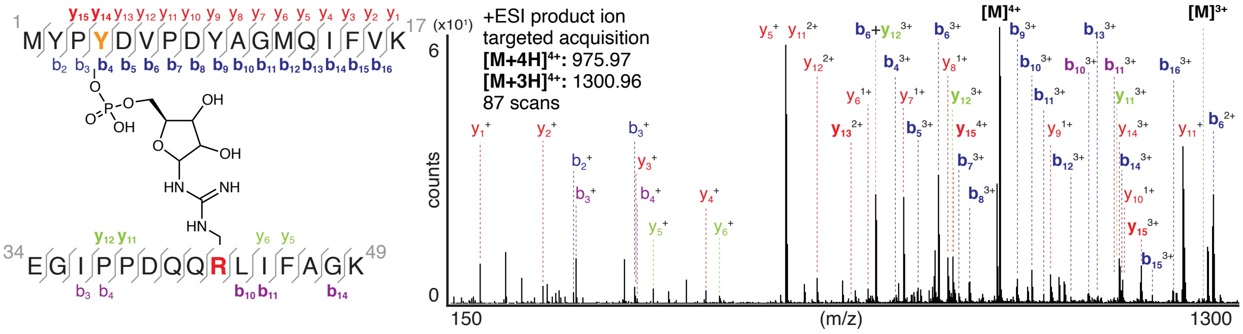

HA tag (1-17) fragmentations:

ion	z	$m/z_{theoretical}$	$m/z_{observed}$	error (amu)	error (ppm)
b_2	1	296.1090	296.1076	-0.0014	-4.73
b_3	1	393.1717	393.1734	0.0017	4.32
b_4	2	1210.0738	1210.0714	-0.0024	-1.98
b_4	3	807.3846	807.3748	-0.0098	-12.14
b_5	2	1267.5873	1267.5795	-0.0078	-6.15
b_5	3	845.3940	845.3875	-0.0065	-7.69
b_6	2	1317.6176	1317.6194	0.0018	1.37
b_6	3	878.0808	878.0810	0.0002	0.23
b_7	3	910.7662	910.7623	-0.0039	-4.28
b_8	3	949.1085	949.1054	-0.0031	-3.27
b_9	3	1003.4645	1003.4618	-0.0027	-2.69
b_{10}	3	1027.1435	1027.1440	0.0005	0.49
b_{11}	3	1046.1506	1046.1446	-0.0060	-5.74
b_{12}	3	1090.1653	1090.1652	-0.0001	-0.09
b_{13}	3	1132.8514	1132.8465	-0.0049	-4.33
b_{14}	3	1170.2117	1170.2082	-0.0035	-2.99
b_{15}	3	1219.2345	1219.2450	0.0105	8.61
b_{16}	3	1252.5917	1252.5903	-0.0014	-1.12

ion	z	$m/z_{theoretical}$	$m/z_{observed}$	error (amu)	error (ppm)
y_{15}	3	1201.9167	1201.9062	-0.0105	-8.74
y_{15}	4	901.6893	901.6833	-0.0060	-6.65
y_{14}	3	1169.8981	1169.8777	-0.0204	-17.44
y_{13}	1	1482.7253	1482.7178	-0.0075	-5.06
y_{13}	2	741.3624	741.3785	0.0161	21.72
y_{12}	1	1367.6984	1367.6971	-0.0013	-0.95
y_{12}	2	684.3506	684.3550	0.0044	6.43
y_{11}	1	1269.6333	1269.6364	0.0031	2.44
y_{11}	2	635.3232	635.3203	-0.0029	-4.56
y_{10}	1	1172.5805	1172.5830	0.0025	2.13
y_9	1	1057.5536	1057.5511	-0.0025	-2.36
y_8	1	893.4869	893.4896	0.0027	3.02
y_7	1	822.4498	822.4543	0.0045	5.47
y_6	1	765.4283	765.4322	0.0039	5.10
y_5	1	634.3879	634.3888	0.0009	1.42
y_4	1	506.3293	506.3335	0.0042	8.29
y_3	1	393.2452	393.2486	0.0034	8.65
y_2	1	246.1768	246.1805	0.0037	15.03
y_1	1	147.1084	147.1108	0.0024	16.31

R42 Tryptic product (34-49) fragmentations:

ion	z	$m/z_{theoretical}$	$m/z_{observed}$	error (amu)	error (ppm)
b_3	1	301.1633	301.1610	-0.0023	-7.64
b_4	1	398.2160	398.2048	-0.0112	-28.13
b_{10}	3	1122.5271	1122.5177	-0.0094	-8.37
b_{11}	3	1160.5468	1160.5504	0.0036	3.10
b_{14}	3	1252.5917	1252.5903	-0.0014	-1.12

ion	z	$m/z_{theoretical}$	$m/z_{observed}$	error (amu)	error (ppm)
y_{12}	4	901.1808	901.1825	0.0017	1.89
y_{11}	3	1169.2222	1169.2103	-0.0119	-10.18
y_6	1	535.3194	535.3090	-0.0104	-19.43
y_5	1	422.2354	422.2308	-0.0046	-10.89

Double N-terminal Pro fragmentation:

ion	z	$m/z_{theoretical}$	$m/z_{observed}$	error (amu)	error (ppm)
b_6+y_{12}	3	778.3633	778.3646	0.0013	1.67

Table S2: Mass Error Analysis for Ub_{WT} + HA Peptide Phenylalanine Variants xl-fragment
Mass error analysis for identified and assigned fragment ions labeled in **Main Text Figure 5**.

ion	peptide	z	$m/z_{theoretical}$	$m/z_{observed}$	error (amu)	error (ppm)
b_2	HA_{WT}	2	1079.5119	1079.508	-0.0041	-3.80
b_2	HA_{Y2F}	2	1071.5144	**undetected**		
b_2	HA_{Y4F}	2	1079.5119	1079.5103	-0.0016	-1.48
b_2	HA_{Y9F}	2	1079.5119	1079.4992	-0.0127	-11.76
y_{11}	HA_{WT}	3	1040.1470	1040.1502	0.0032	3.08
y_{11}	HA_{Y2F}	3	1040.1470	1040.1480	0.0010	0.96
y_{11}	HA_{Y4F}	3	1034.8154	1034.8160	0.0006	0.58
y_{11}	HA_{Y9F}	3	1034.8154	1034.8190	0.0036	3.48
b_6	HA_{WT}	3	878.0808	878.0798	-0.0010	-1.14
b_6	HA_{Y2F}	3	873.0836	873.0877	0.0041	4.70
b_6	HA_{Y4F}	3	873.0836	873.0908	0.0072	8.25
b_6	HA_{Y9F}	3	878.0808	878.0822	0.0014	1.59
y_7	HA_{WT}	3	882.0765	882.0756	-0.0009	-1.02
y_7	HA_{Y2F}	3	882.0765	882.0752	-0.0013	-1.47
y_7	HA_{Y4F}	3	882.0765	882.0752	-0.0013	-1.47
y_7	HA_{Y9F}	3	877.0808	**undetected**		

Table S3: Mass Error Analysis for Ub_{WT} + HA_{Y249S} xl-fragment.
Mass error analysis for identified and assigned fragment ions labeled in **Main Text Figure 6**.

ion	z	$m/z_{theoretical}$	$m/z_{observed}$	error (amu)	error (ppm)
b_2	1	221.0935	221.0910	-0.0025	-11.31
b_3	1	317.1404	317.1359	-0.0045	-14.19
b_4	1	405.1758	405.1754	-0.0004	-0.99
b_5	1	519.1994	519.1908	-0.0086	-16.56
b_5	3	618.2678	618.2570	-0.0108	-17.47
b_{10}	3	951.1122	951.1153	0.0031	3.26
b_{11}	3	970.1193	970.1133	-0.0060	-6.18
b_{12}	3	1014.4684	1014.4676	-0.0008	-0.79
y_{12}	3	1018.4690	1018.4547	-0.0143	-14.04
y_{11}	3	989.4595	989.4663	0.0068	6.87
y_{10}	3	956.4396	956.4321	-0.0075	-7.84
y_9	3	927.4290	927.4353	0.0063	6.79
y_8	3	889.7555	889.7499	-0.0056	-6.29
y_7	3	857.0672	857.0643	-0.0029	-3.38
y_3	1	334.1266	334.1282	0.0016	4.79
y_2	1	276.1018	276.1076	0.0058	21.01

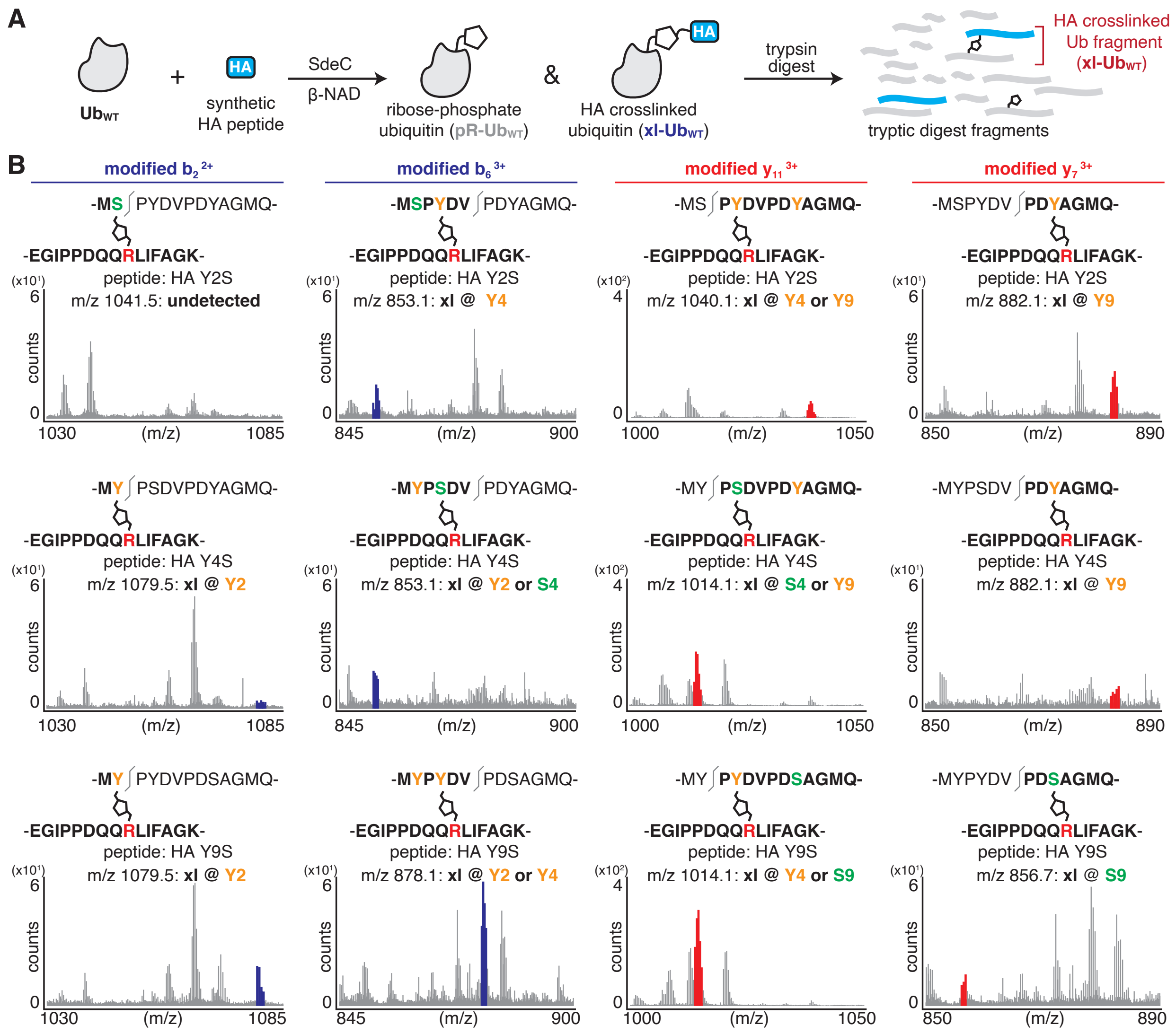

Figure S2. SdeC does not have an intrinsic preference for Ser over Tyr linkages. A) Scheme depicting the SdeC-catalyzed phosphoribosylation of ubiquitin (Ub_{WT}) and its crosslinking to synthetic HA peptides in the presence of β-NAD. **B)** After tryptic digest, peptide mixtures were analyzed using LC-MS/MS. Key fragmentation ions are highlighted, indicating that crosslinking can occur at multiple positions within the synthetic HA peptide. The m/z for the modified b_2 ion, which would suggest crosslinking at Y2 or S2, is absent in HA_{Y2S}, but present in HA_{Y4S} and HA_{Y9S}. The modified b_6 ion is present in all three peptide variants, and indicates crosslinking at Y4 for HA_{Y2S}. For HA_{Y4S} , the presence of this ion suggests that the site of modification may be at either Y2 or S4. For HA_{Y4S}, the presence of this ion suggests that the site of modification may be at either Y2 or Y4. Similarly, a modified y_{11} ion is present in all peptide variants, though this ion could suggest two possible sites of crosslinking, as indicated for each spectra. Finally, the modified y_7 ion indicates crosslinking at Y9 or S9 only. We found that the only definitive evidence of modification at serine for these peptides occurred at S9 for HA_{Y9S}. For HA_{Y2S} and HA_{Y4S}, this same ion is present, but indicates crosslinking occurs at Y9.

Table S4: Mass Error Analysis for Ub_{WT} + HA Peptide Serine Variants xl-fragment. Mass error analysis for identified and assigned fragment ions labeled in **Figure S2**.

ion	peptide	z	$m/z_{theoretical}$	$m/z_{observed}$	error (amu)	error (ppm)
b_2	HA_{WT}	2	1079.5119	1079.5078	-0.0041	-3.80
b_2	HA_{Y2S}	2	1041.4962	**undetected**		
b_2	HA_{Y4S}	2	1079.5119	1079.5188	0.0069	6.39
b_2	HA_{Y9S}	2	1079.5119	1079.5068	-0.0051	-4.72
b_6	HA_{WT}	3	878.0808	878.0798	-0.0010	-1.14
b_6	HA_{Y2S}	3	853.0715	853.0513	-0.0202	-23.68
b_6	HA_{Y4S}	3	853.0715	853.0604	-0.0111	-13.01
b_6	HA_{Y9S}	3	878.0808	878.0679	-0.0129	-14.69
y_{11}	HA_{WT}	3	1040.1470	1040.1502	0.0032	3.08
y_{11}	HA_{Y2S}	3	1040.1470	1040.1452	-0.0018	-1.73
y_{11}	HA_{Y4S}	3	1014.1310	1014.1343	0.0033	3.25
y_{11}	HA_{Y9S}	3	1014.1310	1014.1243	-0.0067	-6.61
y_7	HA_{WT}	3	882.0765	882.0756	-0.0009	-1.02
y_7	HA_{Y2S}	3	882.0765	882.0753	-0.0012	-1.36
y_7	HA_{Y4S}	3	882.0765	882.0596	-0.0169	-19.16
y_7	HA_{Y9S}	3	856.7327	856.7494	0.0167	19.49

Table S5. Plasmids used in this study.

**Plasmids**			
**No.**	**Plasmid Name**	**Details**	**Protein activity**
**1**	pQE-80L-SdeC$_{WT}$[1]	pQE-80L *sdeC*, N-terminus 6XHis tag, C-terminus StrepII tag	SdeC WT
**2**	pQE-80L-SdeC$_{H416A}$[1]	pQE-80L *sdeC* H416A, N-terminus 6XHis tag, C-terminus StrepII tag	SdeC NP-
**3**	pQE-80L-SdeC$_{E859A}$[1]	pQE-80L *sdeC* E859A, N-terminus 6XHis tag, C-terminus StrepII tag	SdeC ART-

Table S6. Peptides used in this study.

**Peptides**			
**No.**	Peptide Name	Sequence	Exact Mass (mono)
**1**	HA$_{WT}$	MYPYDVPDYAGMQ-NH_2	1547.631
**2**	HA$_{Y2F}$	M**F**PYDVPDYAGMQ-NH_2	1531.637
**3**	HA$_{Y4F}$	MYP**F**DVPDYAGMQ-NH_2	1531.637
**4**	HA$_{Y9F}$	MYPYDVPD**F**AGMQ-NH_2	1531.637
**5**	HA$_{Y249F}$	M**F**P**F**DVPD**F**AGMQ-NH_2	1499.647
**6**	HA$_{Y2S}$	M**S**PYDVPDYAGMQ-NH_2	1471.600
**7**	HA$_{Y4S}$	MYP**S**DVPDYAGMQ-NH_2	1471.600
**8**	HA$_{Y9S}$	MYPYDVPD**S**AGMQ-NH_2	1471.600
**9**	HA$_{Y249S}$	M**S**P**S**DVPD**S**AGMQ-NH_2	1319.538

Supplementary References

1. Kotewicz, K. M. *et al.* A single legionella effector catalyzes a multistep ubiquitination pathway to rearrange tubular endoplasmic reticulum for replication. *Cell Host Microbe* **21,** 169–181 (2017).
2. Machner, M. P. & Isberg, R. R. Targeting of host Rab GTPase function by the intravacuolar pathogen Legionella pneumophila. *Dev. Cell* **11,** 47–56 (2006).
3. Khatun, J., Ramkissoon, K. & Giddings, M. C. Fragmentation characteristics of collision-induced dissociation in MALDI TOF/TOF mass spectrometry. *Anal. Chem.* **79,** 3032–3040 (2007).
4. Brenton, A. G. & Godfrey, A. R. Accurate mass measurement: terminology and treatment of data. *J Am Soc Mass Spectrom* **21,** 1821–1835 (2010).
